# Changes in Primary Care Quality Associated With Implementation of the Veterans Health Administration Preventive Health Inventory

**DOI:** 10.1001/jamanetworkopen.2023.8525

**Published:** 2023-04-17

**Authors:** Chelle L. Wheat, Eric J. Gunnink, Jorge Rojas, Ami Shah, Karin M. Nelson, Edwin S. Wong, Kristen E. Gray, Susan E. Stockdale, Ann-Marie Rosland, Evelyn T. Chang, Ashok Reddy

**Affiliations:** 1 Center for Veteran-Centered and Value-Driven Care, Veterans Affairs Puget Sound Health Care System, Seattle, Washington; 2 Office of Primary Care, Veterans Health Affairs, Washington, DC; 3 Department of Health Systems and Population Health, University of Washington, Seattle; 4 Division of General Internal Medicine, Department of Medicine, University of Washington, Seattle; 5 Department of Psychiatry and Biobehavioral Medicine, David Geffen School of Medicine, University of California at Los Angeles; 6 Center for the Study of Healthcare Innovation, Implementation and Policy (CSHIIP), Veterans Affairs Greater Los Angeles Healthcare System, Los Angeles, California; 7 Department of Internal Medicine, University of Pittsburgh, Pittsburgh, Pennsylvania; 8 Center for Health Equity Research and Promotion, Veterans Affairs Pittsburgh Health Care System, Pittsburgh, Pennsylvania; 9 Division of General Internal Medicine, Department of Medicine, David Geffen School of Medicine, University of California at Los Angeles; 10 Division of General Internal Medicine, Department of Medicine, Veterans Affairs Greater Los Angeles Healthcare System, Los Angeles, California

## Abstract

**Importance:**

The COVID-19 pandemic caused significant disruptions in primary care delivery. The Veterans Health Administration (VHA) launched the Preventive Health Inventory (PHI) program—a multicomponent care management intervention, including a clinical dashboard and templated electronic health record note—to support primary care in delivering chronic disease care and preventive care that had been delayed by the pandemic.

**Objectives:**

To describe patient, clinician, and clinic correlates of PHI use in primary care clinics and to examine associations between PHI adoption and clinical quality measures.

**Design, Setting, and Participants:**

This quality improvement study used VHA administrative data from February 1, 2021, through February 28, 2022, from a national cohort of 216 VHA primary care clinics that have implemented the PHI. Participants comprised 829 527 veterans enrolled in primary care in clinics with the highest and lowest decile of PHI use as of February 2021.

**Exposure:**

Templated electronic health record note documenting use of the PHI.

**Main Outcomes and Measures:**

Diabetes and blood pressure clinical quality measures were the primary outcomes. Interrupted time series models were applied to estimate changes in diabetes and hypertension quality measures associated with PHI implementation. Low vs high PHI use was stratified at the facility level to measure whether systematic differences in uptake were associated with quality.

**Results:**

A total of 216 primary clinics caring for 829 527 unique veterans (mean [SD] age, 64.1 [16.9] years; 755 158 of 829 527 [91%] were men) formed the study cohort. Use of the PHI varied considerably across clinics. The clinics in the highest decile of PHI use completed a mean (SD) of 32 997.4 (14 019.3) notes in the electronic health record per 100 000 veterans compared with 56.5 (35.3) notes per 100 000 veterans at the clinics in the lowest decile of use (*P* < .001). Compared with the clinics with the lowest use of the PHI, clinics with the highest use had a larger mean (SD) clinic size (12 072 [7895] patients vs 5713 [5825] patients; *P* < .001), were more likely to be urban (91% vs 57%; *P* < .001), and served more non-Hispanic Black veterans (16% vs 5%; *P* < .001) and Hispanic veterans (14% vs 4%; *P* < .001). Staffing did not differ meaningfully between high- and low-use clinics (mean [SD] ratio of full-time equivalent staff to clinician, 3.4 [1.2] vs 3.4 [0.8], respectively; *P* < .001). After PHI implementation, compared with the clinics with the lowest use, those with the highest use had fewer veterans with a hemoglobin A_1c_ greater than 9% or missing (mean [SD], 6577 [3216] per 100 000 veterans at low-use clinics; 9928 [4236] per 100 000 veterans at high-use clinics), more veterans with an annual hemoglobin A_1c_ measurement (mean [SD], 13 181 [5625] per 100 000 veterans at high-use clinics; 8307 [3539] per 100 000 veterans at low-use clinics), and more veterans with adequate blood pressure control (mean [SD], 20 582 [12 201] per 100 000 veterans at high-use clinics; 12 276 [6850] per 100 000 veterans at low-use clinics).

**Conclusions and Relevance:**

This quality improvement study of the implementation of the VHA PHI suggests that higher use of a multicomponent care management intervention was associated with improved quality-of-care metrics. The study also found significant variation in PHI uptake, with higher uptake associated with clinics with more racial and ethnic diversity and larger, urban clinic sites.

## Key Points

**Question:**

What is the association between adoption of the Veterans Health Administration Preventive Health Inventory (PHI) and primary care clinical quality measures?

**Findings:**

In this quality improvement study that included 829 527 veterans at 216 primary care clinics, clinics with the highest use of the PHI had more veterans with an annual hemoglobin A_1c_ measurement, fewer veterans with a hemoglobin A_1c_ greater than 9% or missing, and more veterans with adequate blood pressure control compared with clinics with low use of the PHI.

**Meaning:**

This study suggests that higher use of a multicomponent care management intervention was associated with improved quality of care.

## Teaser

This quality improvement study describes patient, clinician, and clinic correlates of use of the Veterans Health Administration (VHA) Preventive Health Inventory program in primary care clinics and examines associations between adoption of the program and clinical quality measures.

## Introduction

The COVID-19 pandemic dramatically disrupted primary care delivery, changing how chronic disease care and preventive disease care are provided. Although most excess deaths over the past few years are directly related to COVID-19, up to 40% of deaths are associated with delays in medical care and exacerbation of underlying health conditions.^1,2^ Primary care, which serves as the front line in addressing patients’ preventive and chronic disease care needs, was often postponed or switched from face-to-face care to predominantly telehealth visits.^3,4,5^ Early in the pandemic, these changes in primary care delivery were associated with rates of preventive care screening and medication use decreasing by over 50%.^6,7,8,9,10^ Despite the need for primary care to address delays in needed chronic disease care and preventive care, few systematic interventions have been developed and evaluated to address these needs, to our knowledge.

The Veterans Health Administration (VHA), one of the largest integrated health care systems in the country and serving more than 6 million veterans in more than 1000 primary care clinics, has seen evidence of decreases in primary care services and in quality of care during the COVID-19 pandemic.^11^ The VHA Office of Primary Care (OPC) is focused on mitigating disruptions in preventive care, especially in reference to quality of care.^12^ In this context, the VHA launched a national initiative called the Preventive Health Inventory (PHI) program in February 2021—a multicomponent care management intervention to support primary care in delivering chronic disease care and preventive care that were delayed or disrupted by the pandemic and whose development was informed by the Agency for Healthcare Research and Quality Care Coordination Framework.^13^ First, the initiative took advantage of the VHA electronic health record (EHR) data and informatics systems to create an electronic dashboard of key primary care quality measures to prioritize, accessible to frontline clinicians and clinical operational leaders. Second, the initiative created a templated clinical note—a structured “checklist”—of items to review with veterans that included the following: Columbia Suicide Screening, breast cancer screening, colorectal cancer screening, cervical cancer screening, blood pressure monitoring, diabetes laboratory testing, and retinal screening and foot checks for veterans with diabetes. This templated note is completed by a nurse care manager in primary care via telehealth (either telephone or video). Although the VHA has existing quality measures in primary care that it monitors, the PHI is unique in that it prioritizes some quality measures and guides the primary care team in what measures should be the focus.

The clinical dashboard allows clinicians to easily access multiple sources of information in a concise and usable format and provides them with relevant and timely information needed to improve the quality of patient care.^14^ Templated clinic notes may enforce information standards, reduce documentation time, and provide decision support—all information that feeds back to clinical dashboards. Within the VHA, clinical dashboards and templated notes may encourage collaborative population care among members working on primary care teams and can be used to identify subsets of veterans to target for needed care.^15^ However, existing literature on the individual interventions of clinical dashboards and templated notes are mixed. Although dashboards can improve the clinical efficiency of clinicians, leading to improved care processes and outcomes for patients, there is the potential for harm, including information overload, and existing studies have limitations in their designs that require making cautious conclusions.^16,17^ Furthermore, the association of templated clinical notes with the quality of patient care is also mixed, as standardization may not be as accurate or useful to providing patient care.^18,19,20,21^

The national implementation of the PHI initiative provides a unique opportunity to address knowledge gaps in the potential association with quality of care of a unique intervention that combines clinical dashboards and clinical note templates for care of chronic conditions in primary care. In addition, knowing characteristics that are associated with use of the PHI and whether it improved quality of care, with a focus on the domain of clinical effectiveness, will inform future plans of the OPC. We conducted a quality improvement study with 2 main objectives: (1) to examine patient, clinician, and clinic correlates of PHI use in primary care clinics and (2) to examine associations between adoption of the PHI and quality of care, with a focus on diabetes and hypertension clinical quality measures.

## Methods

### Overview

This is a quality improvement study of VHA clinic sites that used the PHI between February 1, 2021, when OPC rolled out the intervention nationally, and February 28, 2022. We identified the deciles of clinics with the highest and lowest PHI use and compared them to assess characteristics associated with uptake of the PHI. Furthermore, we examined the association between PHI use (high vs low) and monthly clinic quality measures for diabetes and hypertension. The evaluation efforts are part of an ongoing quality improvement effort at the VHA and are not considered research activity as determined by the OPC; thus, they were not subject to institutional review board review or waiver. This study followed the Strengthening the Reporting of Observational Studies in Epidemiology (STROBE) reporting guideline as well as the Standards for Quality Improvement Reporting Excellence (SQUIRE) reporting guideline.

### Data Source

All data for the study were obtained from the VHA Corporate Data Warehouse, a national repository of all clinical and administrative data from the VHA EHR system. One component of the data available from the Corporate Data Warehouse is clinical reminders, which prompt primary care teams about needed care and provide a template for data entry. For this study, we extracted a specific set of clinical reminders that were created to track the implementation and use of the PHI (eMethods in Supplement 1) beginning in February 2021, when the national rollout commenced, and ending in February 2022. Electronic quality measure (eQM) data were extracted from February 2020 to February 2022 to observe trends both before and after PHI implementation. Electronic quality measures are similar to Healthcare Effectiveness Data and Information Set measures.^22^

### Study Sample

The initial cohort for the study was 1020 clinics and approximately 6 million veterans enrolled in primary care. Preventive Health Inventory use was defined as the number of veterans with a documented PHI note in the EHR divided by the total number of veterans enrolled in primary care at the clinic. This number was then converted into a rate per 100 000 veterans and split into deciles to identify the highest and lowest decile of clinics by PHI use. The highest and lowest deciles were chosen to focus on the extremes of the range of PHI use and to clearly highlight any differences in PHI use in terms of the clinical quality metrics. Patients in the study sample included those who were assigned to receive primary care in clinics with the highest and lowest decile of PHI use, respectively, as of February 2021 and through the end of the study period. We excluded veterans who did not have observable data for the entire study period. The final study sample included 108 clinics in each of the high and low categories of use, with a total of 600 097 veterans receiving care in high PHI–use clinics and 229 430 veterans in low PHI–use clinics.

### Outcome Measures

Diabetes and hypertension are 2 of the most common conditions seen among patients in primary care. The OPC regularly monitors performance in these areas to understand the quality of chronic disease management in primary care. Three commonly monitored eQMs for diabetes and hypertension were evaluated separately for their association with PHI use. First, we included the proportion of patients with diabetes with poor control, the numerator for which was the number of veterans between 18 and 75 years of age with a diagnosis of diabetes whose hemoglobin A_1c_ (HbA_1c_) value was greater than 9% (to convert to a proportion of total hemoglobin, multiply by 0.01) or who had no evidence of having their HbA_1c_ measured within the last year. The denominator was all veterans aged 18 to 75 years with a documented diagnosis of diabetes. A lower proportion is considered higher quality. Second, we used the proportion of patients with diabetes who had HbA_1c_ testing completed within the last year, which includes the number of veterans between 18 and 75 years of age with a diagnosis of diabetes who have had HbA_1c_ testing completed within the last year as the numerator and the same denominator as for the poor diabetes control eQM. Last, we examined the proportion of patients with hypertension who had good control, defined as veterans between 18 and 85 years of age with a diagnosis of hypertension with systolic blood pressure of less than 140 mm Hg and diastolic blood pressure of less than 90 mm Hg. The denominator is all veterans aged 18 to 85 years with an active diagnosis of hypertension. A higher proportion is considered higher quality.^5^

### Covariates

Veteran characteristics evaluated included age (years), sex (male or female), race and ethnicity (Alaska Native or American Indian; Asian, Pacific Islander, or Native Hawaiian; Hispanic; non-Hispanic Black; non-Hispanic White; multiple races or ethnicities; or other), marital status (married or other), and Gagne comorbidity score (scores range from <0 to >9, with increased scores corresponding to increased risk of 1-year mortality), all from data recorded in the EHR.^23^ Clinician characteristics included age (years), sex (male or female), panel size (for primary care, measured by number of empaneled patients), full-time equivalent, and tenure at the VHA (years). Clinic size (number of enrolled patients at site), mean staffing ratio (number of support staff for each clinician), facility type for primary care (Veterans Affairs Medical Center [VAMC] or community-based outpatient clinic), and rurality (urban, rural, highly rural, or insular islands) were assessed as facility characteristics. Covariates were obtained from administrative databases (including geographical databases of Rural-Urban Commuting Area codes), and the value from the quarter prior to the date of the last PHI observation (February 28, 2022) was selected.

### Statistical Analysis

Descriptive statistics were calculated using the independent *t* test for continuous variables and the Fisher exact test or the χ^2^ test for categorical variables to evaluate differences in veteran, clinician, and facility characteristics between the highest and lowest deciles of clinics by PHI use. We applied interrupted time series models to estimate changes in diabetes and hypertension eQMs associated with PHI use. The rationale for using the interrupted time series approach was that PHI enactment occurred simultaneously at all VHA primary care clinics in February 2021. As a result, no natural set of comparison sites existed. We stratified by high vs low PHI use at the facility level to evaluate differences in the association between PHI use level and outcomes.

Interrupted time series models used binomial regression to estimate time trends in monthly eQMs for each of the 3 eQMs. The unit of analysis in regression models was the proportion of veterans meeting the criteria for the eQM, weighted by the denominator to obtain the estimated number of veterans conditional on the PHI being used. The primary explanatory variables to capture the association of the PHI was an indicator denoting PHI use, a linear time trend term, and the interaction between these 2 variables. All models were adjusted for veteran race and ethnicity, veteran Gagne comorbidity score, facility rurality, and staffing ratio. The change in eQMs associated with PHI use was calculated for each calendar month after starting use of the PHI. This change was calculated as the difference in eQMs conditional on PHI use and extrapolated eQMs assuming the PHI had not been used. A corresponding 95% CI was calculated for the difference in each month using 1000 bootstrapped replications; 95% CIs that did not cover zero were statistically significant. For each eQM, we summed all monthly differences that were statistically significant, and these were reported as the estimated number of additional veterans (annual HbA_1c_ screening and hypertension control) or fewer veterans (HbA_1c_ >9% or missing) who would have been expected had the PHI not been used.^24^ To convert the results to rates, we took the estimates and divided them by the number of veterans who met the criteria to be included in the eQM denominator for the associated measure and averaged these estimates over the 10 post–PHI period time points. Standard errors from all regression models were heteroskedastic robust. Missing data values for all covariate measures were excluded from the analysis. All statistical analyses were performed using R, version 4.1.1 (R Group for Statistical Computing). All *P* values were from 2-sided tests, and results were deemed statistically significant at *P* < .05.

## Results

### Descriptive Statistics

A total of 1020 clinics caring for approximately 6 million veterans were used to identify clinic use of the PHI. Overall, 829 527 veterans (mean [SD] age, 64.1 [16.9] years; 755 158 of 829 527 [91%] were men) at 216 clinics were included in our evaluation of the highest decile vs the lowest decile of clinics by PHI use. At these clinics, 290 144 veterans received the PHI. The mean (SD) PHI use at the lowest-use clinics was 56.5 (35.3) notes in the EHR per 100 000 veterans compared with 32 997.4 (14 019.3) notes per 100 000 veterans at the highest-use clinics (*P* < .001) (Table). Veteran demographic characteristics differed, with a higher proportion of women at high-use clinics (10% vs 7%; *P* < .001), as well as higher proportions of non-Hispanic Black veterans (16% vs 5%; *P* < .001) and Hispanic veterans (14% vs 4%; *P* < .001).

**Table.** Facility, Clinician, and Patient Characteristics Overall and by Use

Characteristic	Patients, No./total No. (%)		SMD
	Low PHI use (n = 229 430)	High PHI use (n = 600 097)	
Facility characteristics, mean (SD)			
PHI use per 100 000 veterans	56.5 (35.3)	32 997.4 (14 019.3)	−3.3
Clinic size	5712.9 (5825.2)	12 072.0 (7895.3)	−0.92
Staffing ratio	3.4 (0.8)	3.4 (1.2)	0.02
Facility type for primary care			
CBOC	142 468/187 530 (76)	372 769/600 097 (62)	0.30
VAMC	45 062/187 530 (24)	227 328/600 097 (38)	
Facility rurality			
Urban	103 479/180 877 (57)	542 752/596 561 (91)	0.84
Rural	76 072/180 877 (42)	51 165/596 561 (9)	
Highly rural or insular islands	1326/180 877 (1)	2644/596 561 (0.4)	
Patient characteristics			
Age, mean (SD), y	66.1 (16.3)	63.3 (17.1)	0.17
Sex			
Female sex	15 516/229 110 (7)	58 853/598 964 (10)	0.11
Male	213 594/229 110 (93)	540 111/598 964 (90)	
Race and ethnicity			
Alaska Native or American Indian	2584/223 448 (1)	3478/581 603 (0.6)	0.58
Asian, Pacific Islander, or Native Hawaiian	2679/223 448 (1)	9738/581 603 (2)	
Hispanic	7906/223 448 (4)	83 492/581 603 (14)	
Non-Hispanic Black	10 464/223 448 (5)	89 858/581 603 (16)	
Non-Hispanic White	197 003/223 448 (88)	387 324/581 603 (67)	
Multiracial or other	2812/223 448 (1)	7713/581 603 (1)	
Married	107 729/229 430 (47)	243 228/600 097 (41)	0.13
Gagne comorbidity score, mean (SD)	0.3 (1.1)	0.3 (1.2)	−0.05
Clinician characteristics			
Age, mean (SD), y	53.6 (10.2)	53.7 (9.8)	0.00
Sex			
Female	112 113/173 745 (65)	278 156/480 291 (58)	0.14
Male	61 632/173 745 (35)	202 135/480 291 (42)	
Panel size, mean (SD)	877.8 (253.3)	1013.2 (275.7)	−0.51
Full-time equivalent, mean (SD)	0.9 (0.2)	0.9 (0.2)	−0.07
Tenure at VHA, mean (SD), y	10.8 (8.4)	11.7 (7.9)	−0.11

Abbreviations: CBOC, community-based outpatient clinic; PHI, Preventive Health Inventory; SMD, standardized mean difference; VHA, Veterans Health Administration; VAMC, Veterans Affairs Medical Center.

Clinicians at high-use clinics were less likely to be female (58% vs 65%; *P* < .001), had larger mean (SD) panel sizes (1013 [276] veterans vs 878 [253] veterans; *P* < .001), and had a slightly longer mean (SD) tenure at the VHA (11.7 [7.9] years vs 10.8 [ 8.4] years; *P* < .001) (Table). The mean (SD) clinic size was significantly larger at the high-use clinics compared with low-use clinics (12 072 [7895] veterans vs 5713 [5825] veterans; *P* < .001). Although the difference was statistically significant, mean (SD) staffing ratios did not differ meaningfully between high-use and low-use clinics (3.4 [1.2] vs 3.4 [0.8]; standardized mean difference, 0.02; *P* < .001). High-use clinics were more likely to be VAMCs (38% vs 24%; *P* < .001) and located in urban locations (91% vs 57%; *P* < .001).

### Unadjusted Trends in eQMs

**Figure 1.**
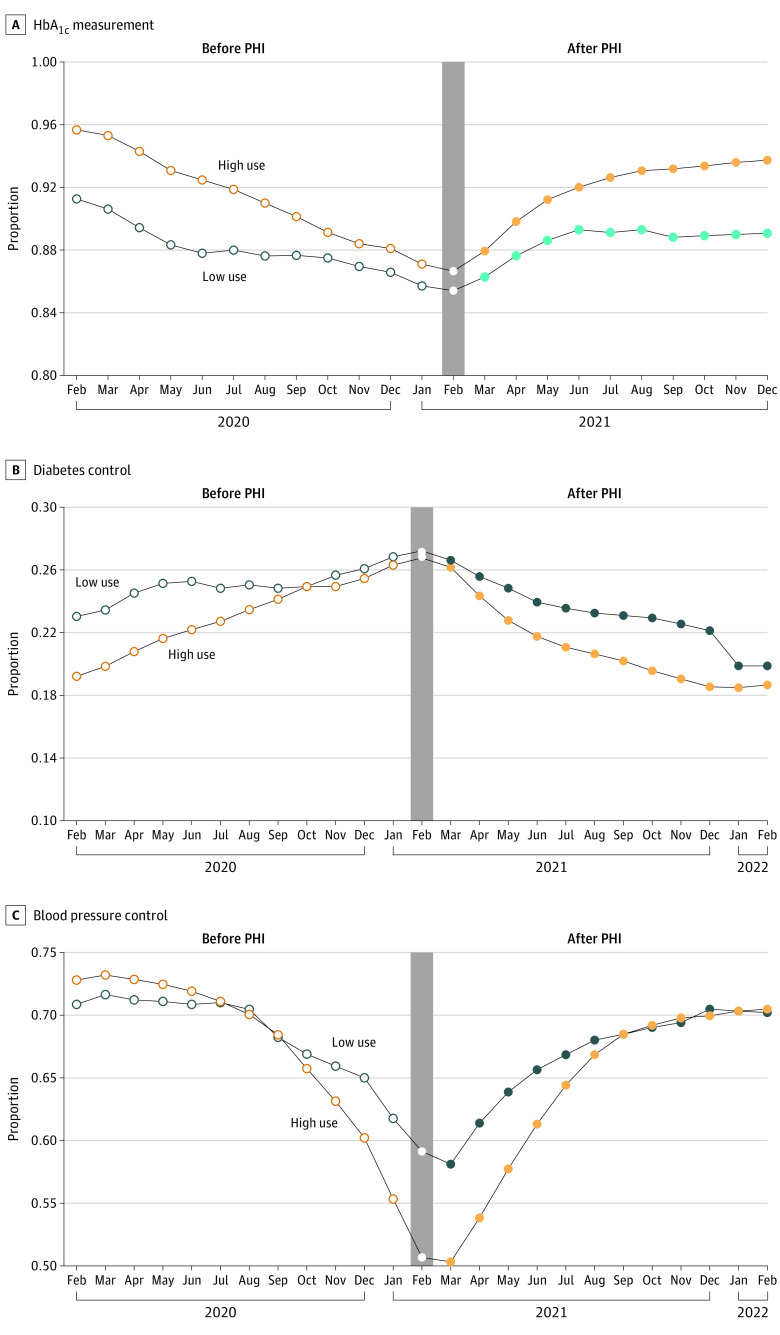
Unadjusted Trends in Diabetes and Hypertension Electronic Quality Measures, February 2020 to February 2022 HbA_1c_ indicates hemoglobin A_1c_; PHI, Preventive Health Inventory.

Monthly unadjusted trends for both diabetes eQMs converge up to PHI enactment and then diverge again, with low-use clinics having a higher proportion than high-use clinics of patients with an HbA_1c_ greater than 9% or missing over the entire study observation period (Figure 1). Annual measurement of HbA_1c_ was greater among high-use clinics compared with low-use clinics across the study period. There was more variability in the blood pressure control eQM. High-use clinics started at a higher proportion of patients with blood pressure control at the beginning of the study observation period (before PHI enactment) but were overtaken by low-use clinics within a few months. That trend continued after PHI enactment until approximately October 2021, when the proportion of patients with blood pressure control at high-use clinics was essentially the same as the proportion at low-use clinics. Overall, our visual interpretation is that the trend in clinical quality of diabetes and hypertension care was decreasing prior to the intervention among both groups and subsequently increased among both groups after the intervention.

### Association of PHI With Diabetes eQMs

**Figure 2.**
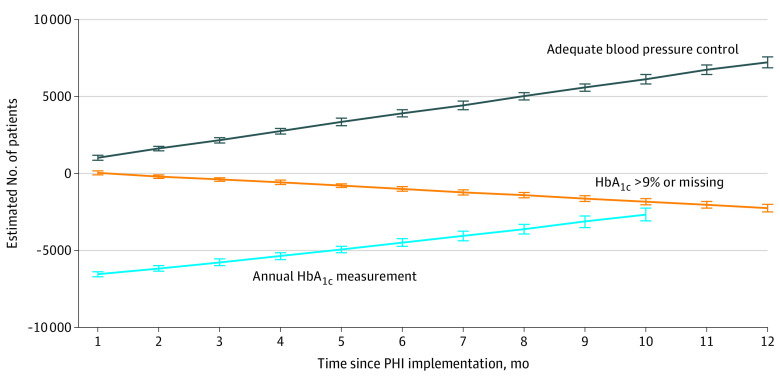
Estimated Trends in Diabetes and Hypertension Electronic Quality Measures After Preventive Health Inventory (PHI) Implementation in Low-Use Clinics HbA_1c_ indicates hemoglobin A_1c_.

**Figure 3.**
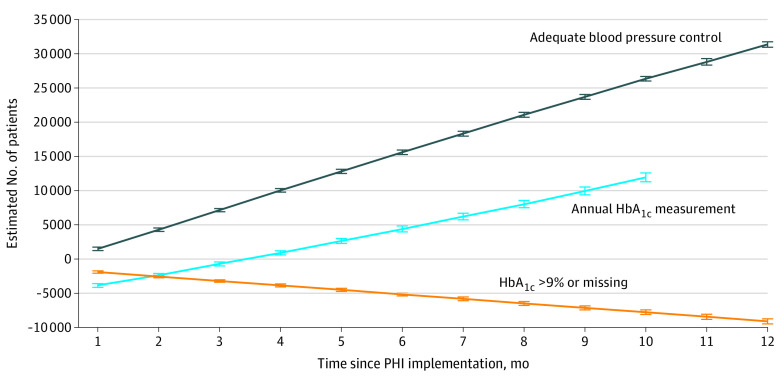
Estimated Trends in Diabetes and Hypertension Electronic Quality Measures After Preventive Health Inventory (PHI) Implementation in High-Use Clinics HbA_1c_ indicates hemoglobin A_1c_.

At the clinics with the lowest use of the PHI, the estimated number of additional veterans having their annual HbA_1c_ measured conditional on the PHI being used was 18 623. In comparison, at the clinics with the highest use of the PHI, an additional 71 823 veterans had their annual HbA_1c_ measured. These estimates translate into mean (SD) rates of 8307 (3539) per 100 000 veterans and 13 181 (5625) per 100 000 veterans for low- and high-use clinics, respectively (Figure 2 and Figure 3, respectively).

At the clinics with the lowest use of the PHI, the estimated number of fewer veterans with an HbA_1c_ greater than 9% or missing conditional on the PHI being used was 16 973. At the clinics with the highest use of the PHI, this number was 61 832 fewer veterans. The corresponding mean (SD) rates were −6577 (3216) per 100 000 veterans at clinics with the lowest use of the PHI and −9928 (4236) per 100 000 veterans at clinics with the highest use of the PHI (Figure 2 and Figure 3, respectively).

### Association of the PHI With the Hypertension eQM

At the clinics with the lowest use of the PHI, the estimated number of additional veterans with adequate blood pressure control conditional on the PHI being used was 44 021. At the clinics with the highest use of the PHI, this number was 202 258 additional veterans. The mean (SD) corresponding rates were 12 276 (6850) per 100 000 veterans at clinics with the lowest use of the PHI and 20 582 (12 201) per 100 000 veterans at clinics with the highest use of the PHI (Figure 2 and Figure 3, respectively).

## Discussion

In this quality improvement study of the VHA’s national implementation of the PHI, we found that higher uptake of the PHI was associated with higher quality of diabetes and hypertension care. Moreover, we found significant variation in uptake of the PHI across over 1000 VHA primary care clinics. Although variation itself is not surprising in the implementation of a large-scale, multicomponent care management intervention, the degree and amount of uptake at 1 year between the clinics with the highest use and the clinics with the lowest use were striking, with 32 997 notes in the EHR per 100 000 veterans and 57 notes per 100 000 veterans, respectively. Our findings provide early evidence suggesting that large-scale primary care interventions can support veterans in catching up from disrupted or delayed care due to the COVID-19 pandemic.

The positive associations that we found between PHI use and 3 commonly tracked quality measures, which was more pronounced for clinics with higher use of the PHI, are similar to the associations between quality and other large-scale, team-based primary care interventions. In the evaluation of the VHA primary care medical home, higher implementation of the primary care medical home was associated with better quality across 48 quality measures.^25,26,27,28,29,30,31^ Other analyses outside of the VHA have shown that care management interventions are associated with reductions in mortality and hospitalization as well as increased rates of annual screening, especially among patients with diabetes.^32,33^ Similarly, care management interventions targeting hypertension have shown significant reductions in blood pressure.^34^ Our study also supports the association between the use of clinical dashboards and templated clinic notes and improved quality of patient care, particularly among team-based primary care models. Clinical dashboards and structured clinic notes obtain and provide information in a concise and usable way that supports primary care clinicians with clinical decision-making.^14,18^ In addition, these interventions encourage collaborative population care across primary care teams and improve the team’s ability to identify and target patients who are in need of chronic disease care or preventive care.^15,16,35^ This evaluation of the PHI initiative addresses knowledge gaps in the association with quality of care of similar interventions within primary care; specifically, it demonstrated that a proactive care management intervention can improve the quality of chronic disease care that has been disrupted by the COVID-19 pandemic.

The VHA OPC is focused on mitigating the disruptions and delayed care due to the pandemic, especially preventive care. Understanding factors associated with variation in use of the PHI as well as the PHI’s association with key quality metrics will assist the OPC in supporting primary care teams in their efforts to provide veterans with needed coordination of chronic disease care and preventive care services. For example, clinics with high use of the PHI were larger and more urban. The PHI builds on existing team-based primary care and informatics systems within the VHA; however, its focus is on virtual delivery of care and is dependent on technology and existing telehealth infrastructure. Larger urban clinics may have technology resources more readily available.^36^ Our findings suggest that additional resources may be needed at smaller and more rural clinics to implement technology-based primary care interventions. Additional research is needed to more fully understand how and why the PHI is being implemented successfully at some sites and not others. This research includes identifying successful implementation strategies, as well as barriers and facilitators to use. Furthermore, understanding individual patient characteristics that may be associated with the PHI’s use is critical.

### Limitations

There are several limitations to this study. First, this is an observational study and can only show associations, not causation. We recognize that our findings may be biased or confounded by other VHA systemwide changes that may be concurrent with the PHI implementation. However, we applied rigorous methods to provide confidence in our estimates. Second, we assessed VHA electronic use of note templates and not direct patient care activities. However, the goal of the PHI note was to better address care that was disrupted due to the pandemic. Third, we used administrative data that may lack clinical information that is captured outside of the VHA or is not available in the EHR system. Fourth, this analysis was conducted at the clinic level and thus does not highlight the individual use of the PHI; however, the PHI was proposed as a clinic-level intervention.

## Conclusions

This quality improvement study suggests that higher use of a multicomponent intervention including clinical dashboards and a templated EHR note was associated with higher-quality diabetes and hypertension care. However, uptake of the intervention varied widely across the national system of more than 1000 primary care clinics. These findings have important implications for the VHA. First, rural and smaller community clinics in the VHA may need additional support to adopt these care coordination tools. Future work should focus on the barriers that these clinics face in implementation of the PHI. Additional resources or facilitation may be needed to support uptake of these tools. Second, multicomponent care management interventions that combine a team-based approach may be associated with improved quality of care. This is especially important to improve chronic disease care that has been disrupted by the COVID-19 pandemic. Third, these results can have important implications for other large, integrated health systems with EHR systems that may be trying to encourage the use of these types of tools for care management and/or coordination.

## Notes

Supplement 1. **eMethods.** HealthFactorType

Supplement 2.
Data Sharing Statement
