# Supplementary material for: Changes in Primary Care Quality Associated With Implementation of the Veterans Health Administration Preventive Health Inventory
Source: JAMA Netw Open. 2023 Apr 17;6(4):e238525. doi: 10.1001/jamanetworkopen.2023.8525 (PMC10111181; doi:10.1001/jamanetworkopen.2023.8525)
Supplement: Supplement 1. — eMethods. HealthFactorType [file jamanetwopen-e238525-s001.pdf]

Supplementary Online Content

Wheat CL, Gunnink EJ, Rojas J, et al. Changes In primary care quality associated with implementation of the Veterans Health Administration Preventive Health Inventory. *JAMA Netw Open.* 2023;6(4):e238525. doi:10.1001/jamanetworkopen.2023.8525

eMethods. HealthFactorType

This supplementary material has been provided by the authors to give readers additional information about their work.

© 2023 Wheat CL et al. *JAMA Network Open.*

eMethods. HealthFactorType
VA-COVID-19 PHI VVC
VA-COVID-19 PHI TELEPHONE
VA-CRC FOLLOW UP DECLINED
VA-CRC AVG RISK ORDER COLONOSCOPY
VA-CRC AVG RISK ORDER FOBT/FIT
VA-DIABETIC FOOT EXAM
VA-COVID-19 PHI DM FOOT UNBL TO INSP
VA-COVID-19 PHI DM FOOT INSP DECL
VA-COVID-19 PHI DM FOOT INSP ABN
VA-COVID 19 PHI DM FOOT INSP NO ABN
WH MAMMOGRAM DECLINED
WH ORDER MAMMOGRAM SCREEN HF
HTN SELF-RECORDED BP
VA-CSSRS DECLINED
WH OUTSIDE NORMAL PAP
WH OUTSIDE ABNORMAL (OTHER) PAP
WH OUTSIDE ABNORMAL (ASCUS) PAP
WH PAP SMEAR DECLINED
WH ORDER PAP SMEAR SCREEN HF
VA-INFLUENZA IMM NONE CURRENT SEASON
VA-FLU EDUCATION VIRTUAL/TELEHEALTH VST
VA-INFLUENZA IMM REFUSED

© 2023 Wheat CL et al. *JAMA Network Open.*
