# Supplementary material for: Changes in Primary Care Quality Associated With Implementation of the Veterans Health Administration Preventive Health Inventory
Source: JAMA Netw Open. 2023 Apr 17;6(4):e238525. doi: 10.1001/jamanetworkopen.2023.8525 (PMC10111181; doi:10.1001/jamanetworkopen.2023.8525)
Supplement: Supplement 2. — Data Sharing Statement [file jamanetwopen-e238525-s002.pdf]

Data Sharing Statement

Wheat. Changes in Primary Care Quality Associated With Implementation of the Veterans Health Administration Preventive Health Inventory. *JAMA Netw Open*. Published April 17, 2023. doi:10.1001/jamanetworkopen.2023.8525

Data
Data available: No
